# The Impact of a Dedicated “Hot List” on the In-Patient Management of Patients With Acute Gallstone-Related Disease

**DOI:** 10.3389/fsurg.2021.643077

**Published:** 2021-05-13

**Authors:** Saurabh Jamdar, Vishnu V. Chandrabalan, Rami Obeidallah, Panagiotis Stathakis, Ajith K. Siriwardena, Aali J. Sheen

**Affiliations:** ^1^Regional Hepato-Pancreato-Biliary Service, Manchester Royal Infirmary, Manchester, United Kingdom; ^2^Faculty of Biology, Medicine and Health, University of Manchester, Manchester, United Kingdom; ^3^Centre of Biosciences, Manchester Metropolitan University, Manchester, United Kingdom

**Keywords:** gallstone, biliary colic, cholecystitis, laparoscopic cholecystectomy, pancreatitis

## Abstract

**Background:** Index admission laparoscopic cholecystectomy is the standard of care for patients admitted to hospital with symptomatic acute cholecystitis. The same standard applies to patients suffering with mild acute biliary pancreatitis. Operating theatre capacity can be a significant constraint to same admission surgery. This study assesses the impact of dedicated theatre capacity provided by a specialist surgical team on rates of index admission cholecystectomy.

**Methods:** This clinical cohort study compares the management of patients with symptomatic gallstone disease admitted to a tertiary care university teaching hospital over two equal but chronologically separate time periods. The periods were before and after service reconfiguration including a specialist HPB service with dedicated operating theatre time allocation.

**Results:** There was a significant difference in the number of admissions over the two time periods with a greater proportion of patients having index admission surgery in the second time period with correspondingly fewer having more than one admission during this latter time period. In the second time period 43% of patients underwent index admission cholecystectomy compared to 23% in the first (*P* < 0.001). The duration of surgery was shorter for patients undergoing surgery during the second time period [135 (102–178) min in the first period and in the second period 106 (89–145) min] (*P* = 0.02).

**Discussion:** This paper shows that the concentration of theatre resources and surgical expertise into regular theatre access for patients undergoing urgent laparoscopic cholecystectomy is an effective and safe model for dealing with acute biliary disease.

## Introduction

Laparoscopic cholecystectomy undertaken during the index admission is the standard of care for patients with acute cholecystitis and also for those with mild or moderate acute biliary pancreatitis (1–3). Index-admission management of symptomatic gallstone disease prevents recurrent biliary events and reduces healthcare costs (4, 5). For such a strategy to be feasible, the necessary surgical expertise must be available to provide this level of care. Acute biliary surgery can be technically demanding and carries risks which can range from a higher proportion of conversion from laparoscopic to open surgery to an increased frequency of bile duct injury. Whilst these risks are faced by all surgeons undertaking this type of surgery, they can be addressed by having surgeons of appropriate expertise and training undertaking these operations (6).

In addition to the necessary surgical expertise, operating theatre time and capacity are often determining factors in whether patients undergo same admission laparoscopic cholecystectomy. Although not an elective procedure, urgent biliary surgery may not be regarded as “life or limb threatening” and therefore may not be prioritised in a mixed access emergency operating theatre system. For example, in a large multicenter prospective audit of patients undergoing cholecystectomy only 16% were operated on as urgencies during the index admission (7).

A potential solution to this problem is the establishment of a dedicated “hot list” for acute biliary surgery staffed by experienced hepato-pancreato-biliary (HPB) surgeons. This study reports on the effect of establishing a thrice weekly list for acute gallbladder surgery on patients undergoing acute laparoscopic cholecystectomy. Outcomes in a 12 month period after establishment of the “hot lists” are compared to results prior to creation of this service when gallbladder surgery was managed as a General Surgical urgency.

## Methods

### Design

This is a clinical cohort study comparing the in-patient management of patients with symptomatic gallstone disease over two equal but chronologically separate time periods.

### Setting

This is a single-centre study set in the General Surgery and Hepato-Pancreato-Biliary Surgery services of the Manchester Royal Infirmary (MRI) a secondary and tertiary care university teaching hospital housing a regional Hepato-pancreato-biliary service which serves a conurbation of 3.2 million.

The first time period is from the 1st January 2013 to the 31st December 2013 during which patients with acute gallstone conditions were admitted to the General surgery service and managed by General, colorectal, and specialist HPB surgeons participating in General on-call. There was no dedicated theatre for patients with gallstone disease and urgent in-patient operations were undertaken on an emergency operating list shared with multiple other surgical specialties. The second time period is 1st January 2015 to the 31st December 2015 following the creation of a regional specialist HPB service at the Manchester Royal Infirmary. Following the establishment of the regional HPB service a 24 h per day/365 days per annum emergency HPB rota was introduced in October 2014 with provision for three dedicated theatre sessions each week for urgent or acute cholecystectomy to be undertaken by surgeons with a special interest in HPB surgery. All patients admitted with suspected acute biliary disease were transferred to the care of this specialist HPB surgical team.

### Patients

Patients admitted between 1st January 2013 and 31st December 2013 constitute the initial cohort and those admitted between 1st January 2015 and 31st December 2015 constitute the second cohort. The hospital medical coding database was searched for all patients admitted at least once as an emergency during the study periods with international classification of Disease (ICD) version 10 codes related to biliary pathology (8). Patients were included if they were over 16 years old, admitted with acute cholecystitis, mild biliary pancreatitis or biliary colic. For the purposes of this study, cholecystitis was defined as acute inflammation of the gallbladder, confirmed by ultrasonographic or computed tomographic evidence. The definition of acute cholecystitis provided by the Tokyo guidelines was used (9). Acute biliary pancreatitis was defined according to the criteria of the 2012 revision of the Atlanta classification (10). Patients admitted as an emergency with obstructive jaundice due to choledocholithiasis who still had their gallbladder *in situ* were also included in the study. Patients were excluded if they were admitted electively or had previously undergone cholecystectomy.

### Data

Data were collected from prospectively maintained electronic databases on demographic details, basis of diagnosis, Charlson co-morbidity index, American Society of Anesthesiology (ASA) grade, length of stay in hospital, and number of re-admissions. Data regarding surgical, endoscopic and radiological interventions were also recorded. For the purposes of this study, the first emergency admission was considered the index admission with all subsequent admissions related to gallstones treated as readmission. Data are presented on time to cholecystectomy from index admission, hospital stay and number of admissions prior to cholecystectomy. Patients who were admitted during either study period but who did not undergo surgery during that year were recorded as undergoing no surgery during study period. Data were collected on pre- and post-operative use of ERCP. Operative detail was recorded on choice of laparoscopic or open routes for surgery, operating time (from first incision to closure), conversion to open surgery, post-operative mortality within the first 30 days after surgery and incidence of major bile duct injury. Outcome data also included length of stay in hospital. No quality of life measurements were undertaken.

### Statistical Analyses

Non-parametric tests were used for analysis. Chi-square tests were used to compare categorical variables. Mann–Whitney and Kruskal–Wallis tests were used to compare the distribution of continuous variables between groups. Analysis was performed using R (R project for statistical computing. www.r-project.org).

A *P* < 0.05 was considered significant.

### Ethical Approval

The Clinical Audit and Risk Management Department of the Central Manchester University Hospitals NHS Foundation Trust regarded this study as an audit and the study was registered as audit 6505.

## Results

### Demographics

Three hundred and one patients constituted the first (2013) study group and 433 the second (2015) time period (2015) (Table 1). There was no significant difference in age, gender or range of clinical diagnoses. Co-morbidity was similar between groups as was American Society of Anaesthesia grade (Table 1).

**Table 1:** Demographic details of patients admitted during the two study periods.

**Variable**	**2013 (*N* = 301)**	**2015 (*N* = 433)**	***p***
Median (range) age in years	54 (37–71)	52 (39–69)	0.962
Gender (female)	193 (64%)	292 (67%)	0.393
Diagnosis	ICD diagnosis available in 298 (99%)	ICD diagnosis available in 420 (96%)	
Cholecystitis	128 (43%)	185 (43%)	0.0122
Biliary colic	75 (25%)	136 (31%)	
Biliary pancreatitis	37 (12%)	48 (11%)	
Choledocholithiasis	58 (19%)	51 (12%)	
Charlson Co-morbidity Index (*n* = 296)	7 (4–13)	7 (4–14)	0.347
ASA Grade (*n* = 329)	2 (1–2)	2 (1–2)	0.226

*ICD 10 coding was available for 298 (99%) of the 2013 cohort and 420 (96%) of the 2015 cohort*.

*ASA, American Society of Anesthesiology*.

### Management of Acute Biliary Disease

There was a significant difference in the number of admissions over the two time periods with a greater proportion of patients having index admission surgery in the second time period with correspondingly fewer having more than one admission during this latter time period (Table 2). There was a significant increase in the use of index admission cholecystectomy during the second period. There was a significant reduction in time from admission to surgery for patients with cholecystitis and for those with biliary pancreatitis (Table 2).

**Table 2:** Management of biliary disease.

	**Time periods 2013**	**Time period 2015**	***p***
**Hospital admissions during study period**			
1	160 (53%)	348 (80%)	< 0.01
2	100 (33%)	69 (16%)	
>2	41 (14%)	16 (4%)	
**Timing of cholecystectomy**			
Index	69 (23%)	188 (43%)	<0.01
Interval	113 (38%)	77 (18%)	
No surgery during study period	119 (40%)	168 (39%)	
**Median (range) time from admission to cholecystectomy in days according to disease type**			
Acute cholecystitis	9 (4–54)	4 (3–7)	<0.001
Biliary colic	41 (7–213)	4 (2–32)	<0.001
Biliary pancreatitis	35 (12–69)	6 (4–9)	<0.001
**Use of ERCP**			
Pre-operative	28 (9%)	21 (5%)	<0.01
Post-operative	16 (5%)	29 (7%)	0.57
**Median (range) duration of in-patient stay in days**			
	8 (4–15)	6 (3–10)	<0.001

*ERCP, endoscopic retrograde cholangiopancreatography*.

**Figure 1:**
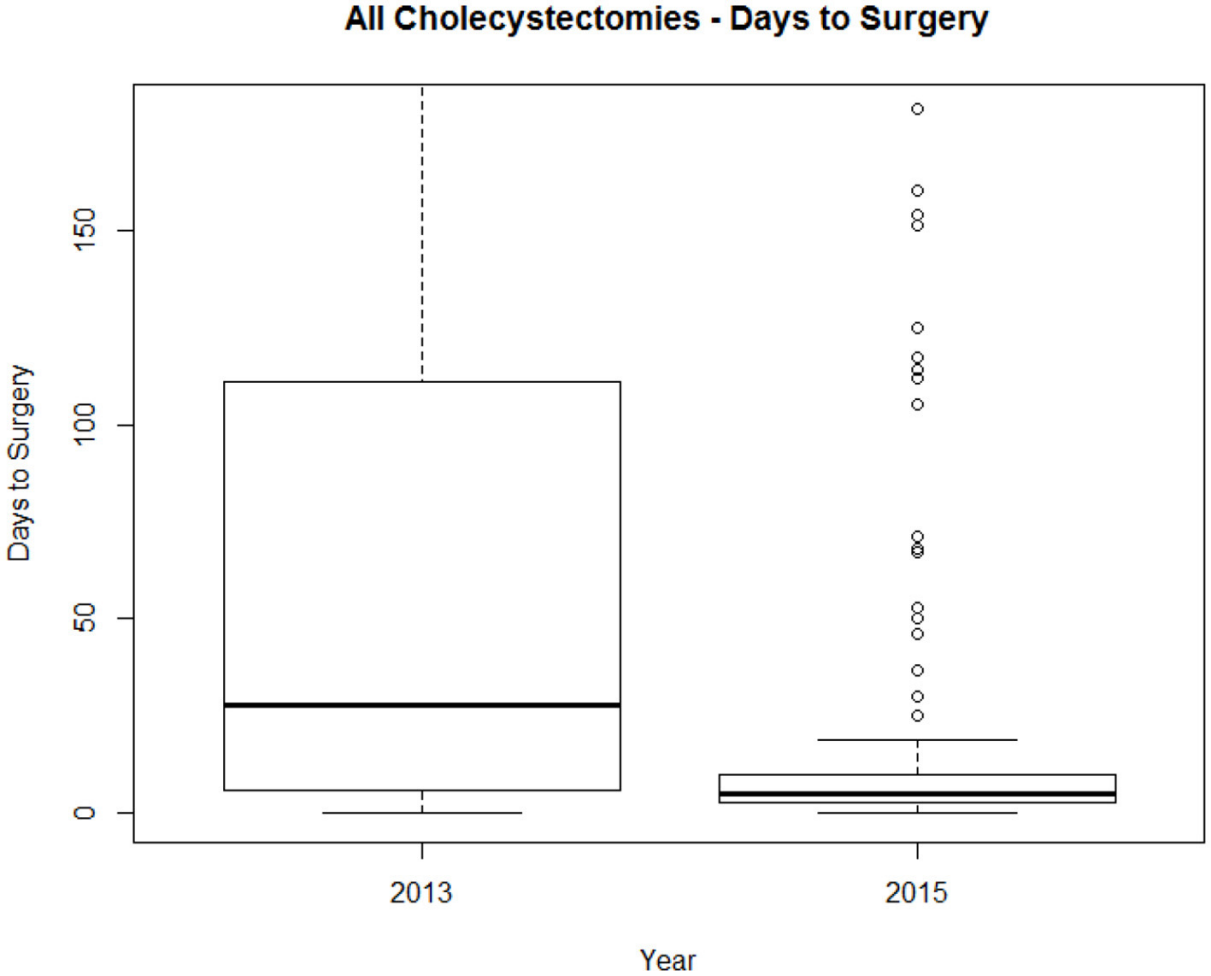
Time to cholecystectomy for entire cohort. Box and Whisker plot showing median time and interquartile range in days to surgery.

There was a significant fall in duration of length of stay between the two time periods. In the second time period 43% of patients underwent index admission cholecystectomy compared to 23% in the first (*P* < 0.001; Figure 1).

### Type of Surgery

In the first time period (2013) 89% of procedures were initiated laparoscopically. In the second time period (2015), 95% were initiated laparoscopically. This difference was not significant (*P* = 0.41). The conversion rate in the first period was 6 and 3% in the second. The duration of surgery in the first period was 135 (102–178) min and in the second period was 106 (89–145). This difference was significant (*P* = 0.02).

### Outcomes

There were no deaths within 30 days of surgery or bile duct injuries after cholecystectomy in either time period.

## Discussion

This paper reports on the effect on cholecystectomy practise of a dedicated “hot list” for acute gallbladder surgery staffed by hepatobiliary surgeons in a large tertiary care acute provider hospital.

The data show that a relatively large number of patients presented for treatment in both study periods. As the population and demographic profile would not have been expected to change materially between the two time periods the reasons for the greater patient numbers in the second time cohort are largely speculative but may relate to “drift” of patients towards tertiary care provider institutions. There was no difference in co-morbidity profile between the two time periods.

The management of acute biliary disease seems to be different between the two time periods with more patients undergoing definitive intervention during the index admission and with a shorter time to surgery. To present a balanced perspective, it should be acknowledged that there were quite prolonged waiting times for surgery for biliary colic and biliary pancreatitis in the 2013 cohort (Table 2). This does provide a potential source of bias.

This greater use of definitive surgery during the index admission translates both to a shorter in-patient stay and a fewer number of re-admissions.

How can these findings be interpreted in a wider healthcare context? It is important to bear in mind that the study was undertaken in the United Kingdom's National Health Service. In the US the acute care surgery model has demonstrated the feasibility of this type of service to deliver high volume emergency cholecystectomies (11). Thus a “hot list” is only necessary if access to operating theatre time for acute biliary surgery is limited. This ease of access will vary globally across different healthcare providers and in settings where there is timely provision for acute biliary surgery, such a “hot list” may not be necessary. The additional theatre time provided by the “hot list” ensured that patients with other surgical emergencies did not have to wait longer for intervention. In healthcare systems with limited access to emergency theatre time, the provision of a hot list within office hours can ensure that patients with acute gallstone diseases can be treated in a timely manner. Secondly, the question of appropriate surgical expertise is relevant. In this report, biliary surgery was undertaken by General Surgeons, Colorectal Surgeons, and specialist HPB surgeons in the first time period and seems to have been effectively managed albeit with a low uptake of index admission surgery and a use of the option to defer. In the second time period, all acute biliary surgery was managed by specialist HPB surgeons. A cause-effect association cannot be assumed here and the reality is that surgical experience especially in the management of acute biliary disease is probably the most important factor. This may be more available in the hands of specialist HPB surgeons but the argument of whether acute biliary surgery should be done by specialists or generalists is a long-running debate and cannot be simply resolved. In reality, the availability of appropriate surgical expertise depends substantially on healthcare resource. It could also be argued that specialist HPB surgeons are more gainfully employed in surgical oncology and that well-trained General Surgeons should be able to treat acute biliary disease.

In conclusion, this paper shows that the concentration of theatre resource and surgical HPB expertise into regular hot lists is an effective and safe model for dealing with acute biliary disease.

## Author Contributions

All the authors were involved from the inception of the hypothesis, collection of data with its subsequent analysis as well as drafting of the manuscript. This data was presented at two local national surgical meetings.

## Conflict of Interest

The authors declare that the research was conducted in the absence of any commercial or financial relationships that could be construed as a potential conflict of interest.

## References

[1] Wiggins T Markar SR MacKenzie H Faiz O Mukherjee D Khoo DE. Optimum timing of emergency cholecystectomy for acute cholecystitis in England: population-based cohort study. Surg Endosc. (2019) 33:2495–2502. 10.1007/s00464-018-6537-x 30949811 PMC6647372

[2] Gurusamy K. Early laparoscopic cholecystectomy appears better than delayed laparoscopic cholecystectomy for patients with acute cholecystitis. Evid Based Med. (2016) 21:28. 10.1136/ebmed-2015-110332 26645472

[3] Banks PA Bollen TL Dervenis C Gooszen HG Johnson CD Sarr MG. Classification of acute pancreatitis−2012: revision of the Atlanta classification and definitions by international consensus. Gut. (2013) 62:102–11. 10.1136/gutjnl-2012-302779 23100216

[4] Seager A Hall TC Dennison AR Garcea G. Economic implications of providing emergency cholecystectomy for all patients with biliary pathology: a retrospective analysis. Surg Laparosc Endosc Percutan Tech. (2015) 25:337–42. 10.1097/SLE.0000000000000169 26121547

[5] Kohga A Suzuki K Okumura T Yamashita K Isogaki J Kawabe A. Outcomes of early versus delayed laparoscopic cholecystectomy for acute cholecystitis performed at a single institution. Asian J Endosc Surg. (2019) 12:74–80. 10.1111/ases.12487 29611896

[6] Bokhari S Walsh U Qurashi K Liasis L Watfah J Sen M. Impact of a dedicated emergency surgical unit on early laparoscopic cholecystectomy for acute cholecystitis. Ann R Coll Surg Engl. (2016) 98:107–115. 10.1308/rcsann.2016.0049 26673047 PMC5210487

[7] CholeS Study Group and West Midlands Research Collaborative. Population-based cohort study of variation in the use of emergency cholecystectomy for benign gallbladder diseases. Br J Surg. (2016) 103:1716–26. 10.1002/bjs.10288 27748962

[8] WHO. ICD-10: International Statistical Classification of Diseases and Health Related Problems—Tenth Revision. Available online: https://www.who.int/standards/classifications/classification-of-diseases (accessed 11th July 2020).

[9] Takada T. Tokyo guidelines 2018: updated Tokyo guidelines for the management of acute cholangitis/acute cholecystitis. J Hepatobiliary Pancreat Sci. (2018) 25:96–100. 10.1002/jhbp.526 29090868

[10] Working Group IAP/APA Acute Pancreatitis Guidelines. IAP/APA evidence-based guidelines for the management of acute pancreatitis. Pancreatology. (2013). 13 (4 Suppl. 2):e1–15. 10.1016/j.pan.2013.07.063 24054878

[11] Fletcher E Seabold E Herzing K Markert R Gans A Ekeh AP. Laparoscopic cholecystectomy in the acute care surgery model: risk factors for complications. Trauma Surg Acute Care Open. (2019) 4:e000312. 10.1136/tsaco-2019-000312 31565675 PMC6744070

